# A murine model demonstrates capsule-independent adaptive immune protection in survivors of *Klebsiella pneumoniae* respiratory tract infection

**DOI:** 10.1242/dmm.043240

**Published:** 2020-03-26

**Authors:** Joy Twentyman, Catherine Morffy Smith, Julia S. Nims, Aubree A. Dahler, David A. Rosen

**Affiliations:** 1 Department of Pediatrics, Division of Pediatric Infectious Diseases, Washington University School of Medicine, St Louis, MO 63110, USA; 2 Department of Molecular Microbiology, Washington University School of Medicine, St Louis, MO 63110, USA

**Keywords:** *Klebsiella pneumoniae*, Adaptive immunity, Murine model, Pneumonia, Capsule

## Abstract

*Klebsiella pneumoniae* represents a growing clinical threat, given its rapid development of antibiotic resistance, necessitating new therapeutic strategies. Existing live-infection models feature high mortality rates, limiting their utility in the study of natural adaptive immune response to this pathogen. We developed a preclinical model of pneumonia with low overall mortality, in which previously exposed mice are protected from subsequent respiratory tract challenge with *K. pneumoniae*. Histologic analyses of infected murine lungs demonstrate lymphocytic aggregates surrounding vasculature and larger airways. Initial exposure in RAG1 knockout mice (lacking functional B and T cells) failed to confer protection against subsequent *K. pneumoniae* challenge. While administration of isolated *K. pneumoniae* capsule was sufficient to provide protection, we also found that initial inoculation with *K. pneumoniae* mutants lacking capsule (Δ*cps*), O-antigen (Δ*wecA*) or both conferred protection from subsequent wild-type infection and elicited *K. pneumoniae*-specific antibody responses, indicating that non-capsular antigens may also elicit protective immunity. Experiments in this model will inform future development of multivalent vaccines to prevent invasive *K. pneumoniae* infections.

## Abstract (precis)

**Summary:** This novel mouse model of nonlethal pulmonary *Klebsiella pneumoniae* infection allows for the exploration of mechanisms required to mount a protective memory response to *K. pneumoniae* in the lung.

## INTRODUCTION

The opportunistic pathogen *Klebsiella pneumoniae* is widespread in the environment and can asymptomatically colonize the human gastrointestinal tract and other mucosal surfaces (Fung et al., 2012; Gorrie et al., 2017; Kock et al., 2016; Lin et al., 2012; Martin and Bachman, 2018; Podschun and Ullmann, 1998; Struve and Krogfelt, 2004). Over the past two decades, the emergence of antibiotic resistance determinants, including *K. pneumoniae* carbapenemases and extended-spectrum beta lactamases (Munoz-Price et al., 2013; Paterson et al., 2004; Santino et al., 2013), make this pathogen an increasingly severe clinical threat. By 2030, the global prevalence of third-generation cephalosporin and carbapenem resistance in *K. pneumoniae* infections is projected to exceed 50% (Alvarez-Uria et al., 2018). Owing to its carriage in the human population, pervasiveness in healthcare settings, and rise in antibiotic resistance, *K. pneumoniae* is responsible for a growing proportion of nosocomial infections, including pneumonia, urinary tract infection and sepsis (David et al., 2019; Podschun and Ullmann, 1992; Tsay et al., 2002). Vaccination or other immunotherapies may prove to be critical tools in the prevention or treatment of *K. pneumoniae* infections in the looming absence of effective antibiotics. Despite the urgency of this threat, no licensed *K. pneumoniae* vaccine is currently available, and vaccine development is hindered by our minimal knowledge of the immune responses to this pathogen.

Murine studies focused on the host immune response to *K. pneumoniae* have largely utilized model isolates (e.g. ATCC 43816) that are highly and rapidly lethal in mice (Fodah et al., 2014; Hsieh et al., 2013; Lau et al., 2007; Lavender et al., 2004; Lawlor et al., 2005; Lin et al., 2014; Wu et al., 2009), precluding their utility in illuminating natural adaptive immune responses to live *K. pneumoniae*. Instead, experiments have assessed adaptive immunity elicited by heat-killed organisms and a variety of specific *K. pneumoniae* immunogenic factors, including outer membrane vesicles, O-antigens, type 3 fimbriae and purified capsule (Chen et al., 2011; Cryz et al., 1985; Lee et al., 2015; Amezcua Vesely et al., 2019; Lavender et al., 2005; Trautmann et al., 2004; Hegerle et al., 2018). Early work in rodents and subsequently humans indicated that immunization with capsule elicits serotype-specific antibody responses (Cryz et al., 1985; Alcantar-Curiel et al., 1993; Cryz et al., 1988, 1984, 1986a,b); however, capsule is not the sole driver of protective immunity during infection (Lee et al., 2015; Lundberg et al., 2013). Moreover, it is currently unknown whether invasive infection in patients elicits durable protective immunity. If so, the host cell types imperative for this protection and the bacterial antigens responsible need to be identified.

To begin assessing these questions, we created a preclinical murine model of survivable *K. pneumoniae* lung infection, followed by subsequent re-challenge, in order to study the development of adaptive immune responses. We found that the majority of mice surviving initial infection with *K. pneumoniae* in the respiratory tract were protected from subsequent challenge in an adaptive immune-dependent manner. We further found that inoculation with live bacteria confers greater protection than inoculation with heat-killed organisms and that *K. pneumoniae* capsule, while an important immune stimulus, is not the sole antigen capable of eliciting protection.

## RESULTS

### Survivors of *K. pneumoniae* respiratory tract infection are protected from subsequent infection

**Fig. 1.**
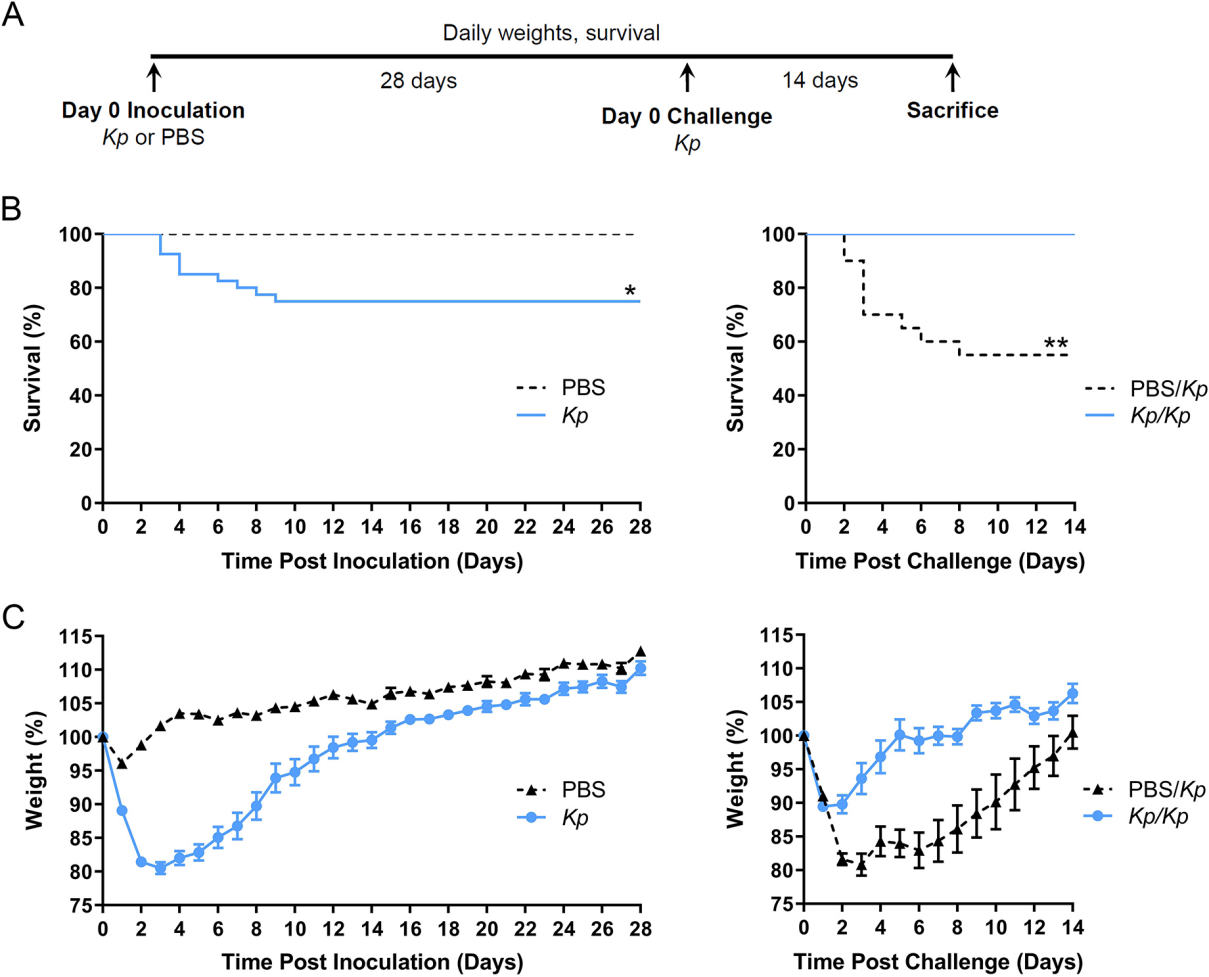
**Survivors of *K. pneumoniae* (*Kp*) respiratory tract inoculation are protected upon subsequent *Kp* challenge.** (A) Schematic of experimental course in which C57BL/6J mice are intratracheally inoculated with *Kp* or PBS followed by challenge with *Kp* 28 days later. (B) Survival of mice inoculated with *Kp* or PBS over 28 days (left; *Kp n*=40, PBS *n*=20) and over 14 days after challenge with *Kp* (right; *Kp/Kp n*=15, PBS*/Kp n*=20). (C) Daily weights of mice inoculated with *Kp* or PBS over 28 days (left) and over 14 days after challenge with *Kp* (right) are presented as a percentage of weight at day 0 inoculation (left) or day 0 challenge (right). Data are shown as mean±s.e.m. and are combined from at least three independent experiments. *P<0.05, **P<0.01; Mantel–Cox log-rank test.

Our prior studies have introduced the *K. pneumoniae* strain TOP52, which causes reproducible experimental pneumonia with low overall lethality (Rosen et al., 2015). Here, we leveraged this model to elucidate whether survivors of initial inoculation would be protected from subsequent *K. pneumoniae* challenge. Female C57BL/6J mice were intratracheally inoculated with 10^7^ colony-forming units (CFU) of *K. pneumoniae* TOP52 or sterile PBS, and weights and survival were followed for 28 days. Surviving mice were subsequently challenged intratracheally with 10^7^ CFU of *K. pneumoniae* TOP52 and monitored for an additional 14 days prior to sacrifice (Fig. 1A). Following initial inoculation, all the mice that received intratracheal PBS survived for 28 days, whereas 75% of *K. pneumoniae*-inoculated mice survived (*P*=0.0170; Fig. 1B). Challenge of both groups of surviving mice with *K. pneumoniae* resulted in 45% mortality in mice that initially had received PBS and no deaths in the *K. pneumoniae*-survivor mouse group (*P*=0.0033; Fig. 1B). We speculate that the increase in mortality associated with TOP52 infection in the PBS/*K. pneumoniae* challenge group relative to the mortality of mice in the initial TOP52 inoculation group is likely related to the difference in age of the mice upon first TOP52 infection and the second invasive surgical procedure in the group with increased mortality.

We measured the weights of mice throughout the experiment as an indicator of overall morbidity after infection. Mice initially inoculated with *K. pneumoniae* had significantly lower weights than PBS-inoculated controls from days 1-25 post-inoculation (Fig. 1C). However, after challenging all survivors with *K. pneumoniae*, those mice originally exposed to *K. pneumoniae* had significantly less weight loss from days 2-12 post-challenge compared to control mice (Fig. 1C). Together, these data demonstrate that, compared to naïve mice, survivors of initial *K. pneumoniae* TOP52 infection exhibit reduced morbidity and mortality upon secondary challenge with *K. pneumoniae* TOP52.

### The *K. pneumoniae* protection phenotype requires the adaptive immune system

**Fig. 2.**
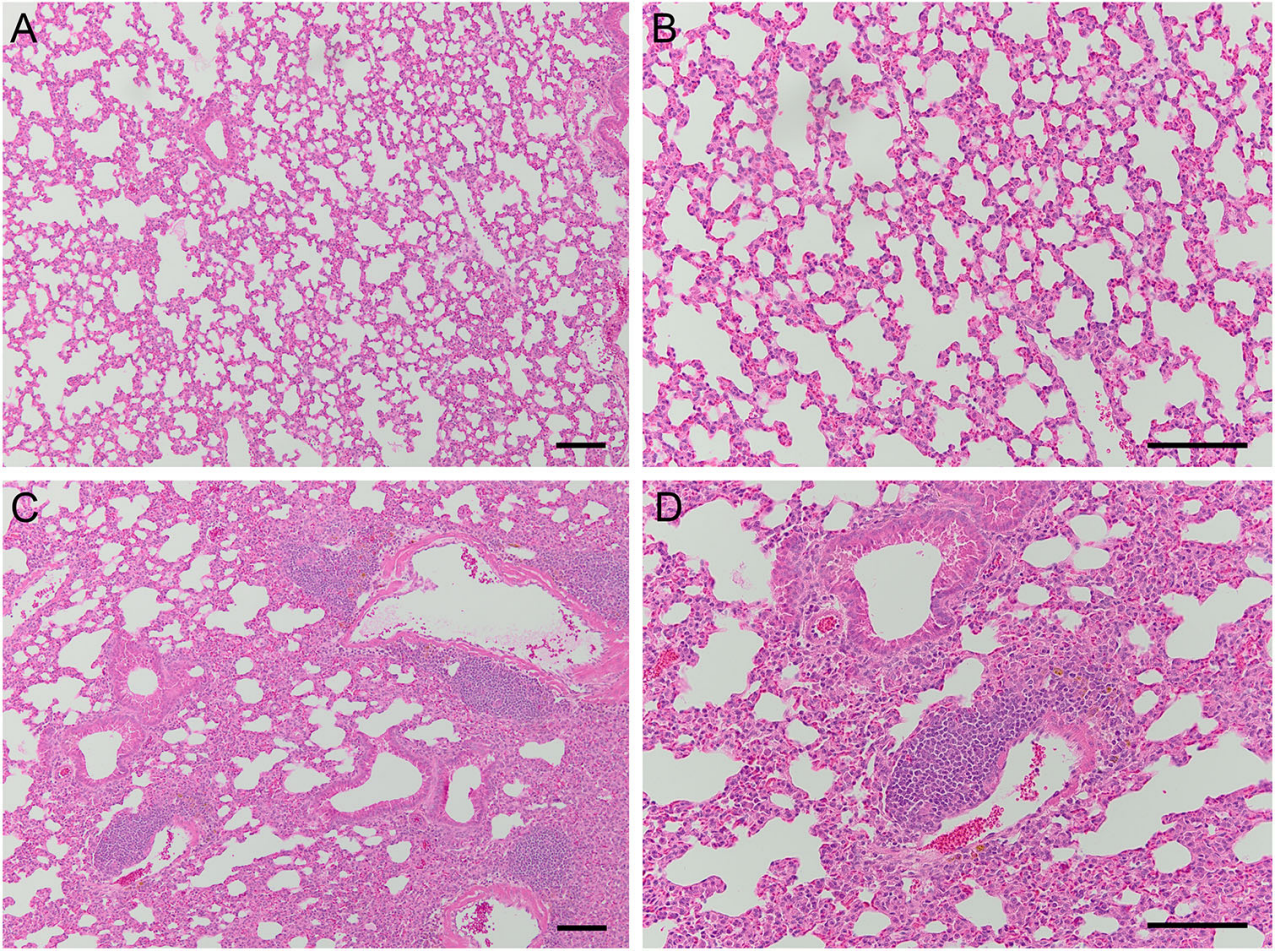
**Mice inoculated with *K. pneumoniae* develop lymphoid aggregates in their lungs.** (A-D) Representative histologic images of lungs harvested 28 days post-inoculation with PBS (A,B) or *K. pneumoniae* TOP52 (C,D) demonstrate a persistent inflammatory infiltrate in TOP52-inoculated organs, including the presence of perivascular lymphocytic collections. Low-power images (A,C) were taken with a 10× objective and higher-power images (B,D) were taken with a 20× objective. Scale bars: 100 µm.

To ensure that the protective phenotype observed was not associated with persistent bacterial infection in *K. pneumoniae*-infected mice, we harvested spleens and lungs 28 days post-inoculation with *K. pneumoniae* or PBS control, prior to challenge. We found that surviving mice cleared the original inoculum, as lungs and spleen had extremely low or undetectable bacterial colonization (Fig. S1A). Histologic examination of lungs at 28 days post-inoculation with *K. pneumoniae* revealed a moderate inflammatory infiltrate not found in PBS-inoculated lungs (Fig. 2). Additionally, we observed perivascular collections of lymphoid cells in most *K. pneumoniae*-inoculated lungs (Fig. 2C,D) that were not present in PBS-inoculated lungs. Of note, mice that survived subsequent challenge with *K. pneumoniae* similarly harbored low or absent bacterial burden in lungs and spleens at 14 days post-challenge (Fig. S1B).

**Fig. 3.**
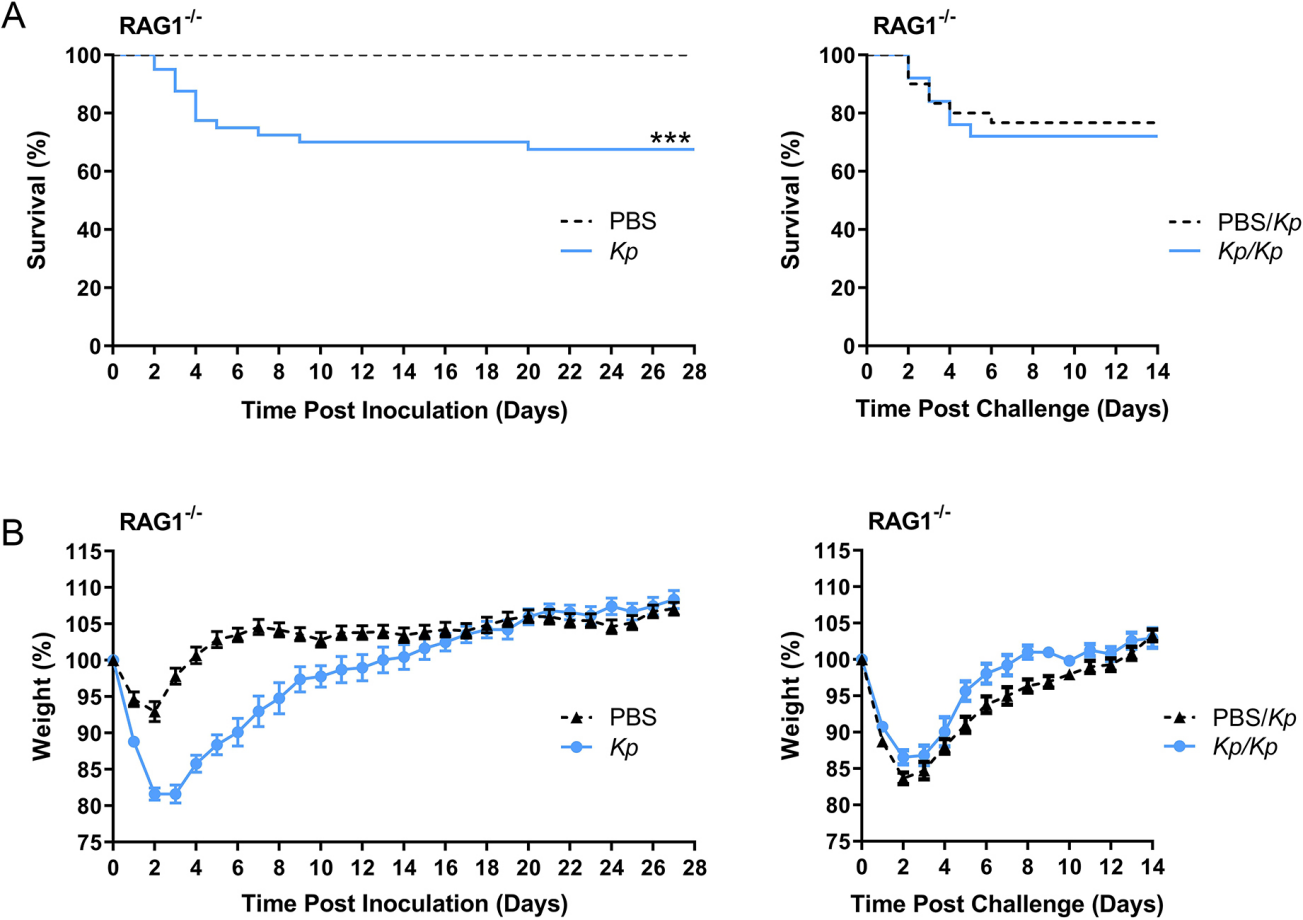
**Survivors of *Kp* respiratory tract inoculation are not protected upon subsequent *Kp* challenge in RAG1^−/−^ mice.** (A) Survival of RAG1^−/−^ mice inoculated with *Kp* or PBS over 28 days (left; *Kp n*=27, PBS *n*=31) and over 14 days after challenge with *Kp* (right; *Kp/Kp n*=25, PBS*/Kp n*=30). (B) Daily weights of RAG1^−/−^ mice inoculated with *Kp* or PBS over 28 days (left) and over 14 days after challenge with *Kp* (right). Data are shown as mean±s.e.m. and are combined from at least three independent experiments. ***P<0.001; Mantel–Cox log-rank test.

To demonstrate that the observed protective phenotype upon challenge with *K. pneumoniae* arose from a host adaptive immune mechanism, we performed analogous experiments in RAG1^−/−^ mice, which lack functional B and T cells. All of the RAG1^−/−^ mice that received intratracheal inoculation with PBS survived for 28 days, whereas 68% of *K. pneumoniae*-inoculated RAG1^−/−^ mice survived (*P*<0.0001; Fig. 3A). As expected, these rates were similar to those rates observed in initial infection of wild-type mice (Fig. 1B), indicating that the innate immune response is sufficient to control initial infection in a majority of, but not all, mice.

Challenge of both groups of surviving RAG1^−/−^ mice with *K. pneumoniae* resulted in similar mortality in the PBS and *K. pneumoniae*-survivor mouse groups (23% and 28% mortality, respectively; Fig. 3A). RAG1^−/−^ mice initially inoculated with *K. pneumoniae* had significantly lower weights than PBS-inoculated control mice from days 1-10 post-inoculation (Fig. 3B). However, after subsequent *K. pneumoniae* challenge, RAG1^−/−^ mice originally exposed to *K. pneumoniae* had weights similar to those of control mice, with the exception of slightly higher weights on days 8-9 (Fig. 3B). Additionally, organs collected from surviving RAG1^−/−^ mice at 14 days post-challenge demonstrated equivalent, low bacterial burdens in both *K. pneumoniae*-exposed and naïve mice (Fig. S2). Together, these experiments demonstrate that the *K. pneumoniae* protection we observed in wild-type mice is lost in RAG1^−/−^ mice, indicating an adaptive immune mechanism of protection.

### Inoculation with heat-killed *K. pneumoniae* provides an intermediate level of protection

**Fig. 4.**
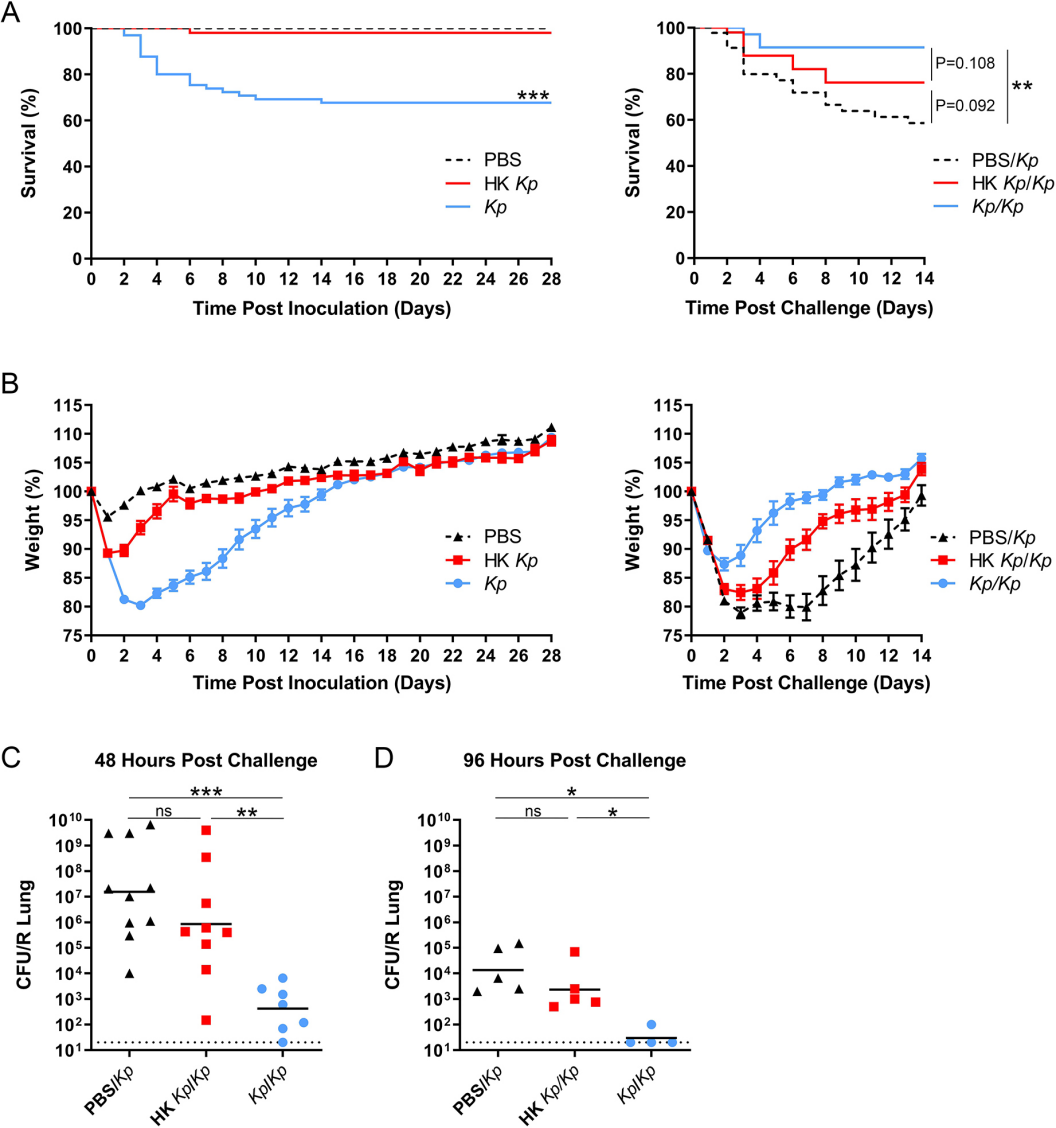
**Heat-killed *K. pneumoniae* (HK *Kp*) respiratory tract inoculation provides an intermediate level of protection upon subsequent *Kp* challenge.** (A) Survival of mice inoculated with *Kp*, HK *Kp* or PBS over 28 days (left; *Kp n*=65, HK *Kp n*=50, PBS *n*=48) and over 14 days after challenge with *Kp* (right; *Kp/Kp n*=35, HK *Kp/Kp n*=39, PBS*/Kp n*=38). (B) Daily weights of mice inoculated with *Kp*, HK *Kp* or PBS over 28 days (left) and over 14 days after challenge with *Kp* (right). Weight data are shown as mean±s.e.m. (C) Lung bacterial titers 48 h post-challenge with *Kp*. (D) Lung bacterial titers 96 h post-challenge. All data are combined from at least three independent experiments. For titers, short bars represent geometric means, and full dotted horizontal lines represent limits of detection. ns, not significant; *P<0.05, **P<0.01, ***P<0.001.

Past studies have used respiratory tract inoculation of heat-killed *K. pneumoniae* or extracellular vesicles to study adaptive protection, likely because infection with live organisms resulted in lethality (Chen et al., 2011; Lee et al., 2015). We next asked whether different levels of protection resulted from exposure to heat-killed versus live *K. pneumoniae*. We inoculated mice with 10^7^ CFU of *K. pneumoniae* TOP52, the equivalent amount of heat-killed *K. pneumoniae* or sterile PBS control. Weights and survival were tracked for 28 days prior to challenge with live *K. pneumoniae*. All of the mice that were inoculated with PBS and 98% of the mice that received heat-killed *K. pneumoniae* survived 28 days post-inoculation, compared to 68% of those initially inoculated with live *K. pneumoniae* (*P*<0.0001 for both comparisons; Fig. 4A). After subsequent challenge with live *K. pneumoniae*, mice previously exposed to heat-killed bacteria demonstrated an intermediate level of protection between live *K. pneumoniae*-exposed mice and *K. pneumoniae*-naïve mice (Fig. 4A).

Mice initially inoculated with heat-killed *K. pneumoniae* had significantly lower weights than PBS control mice (days 1-9 post-inoculation) but significantly higher weights than mice inoculated with live *K. pneumoniae* (on days 2-14 post-inoculation) (Fig. 4B). After challenge with live *K. pneumoniae*, mice originally exposed to heat-killed *K. pneumoniae* had significantly lower weights than live *K. pneumoniae* survivors (on days 2-12 post-challenge) but significantly higher weights than PBS control mice (on days 6-9 post-challenge) (Fig. 4B).

Subsets of mice from this experiment were sacrificed at 48 h or 96 h post-*K. pneumoniae* challenge. Lungs from mice originally inoculated with heat-killed organisms or PBS control demonstrated higher bacterial titers at both 48 h (Fig. 4C) and 96 h (Fig. 4D) post-challenge, relative to mice that originally survived inoculation with live *K. pneumoniae*. Taken together, these data suggest that exposure to heat-killed bacteria provides modest protection from subsequent live challenge but does not confer the full protection evident in mice that survive live *K. pneumoniae* infection.

### Isolated capsule provides protection from subsequent *K. pneumoniae* challenge

**Fig. 5.**
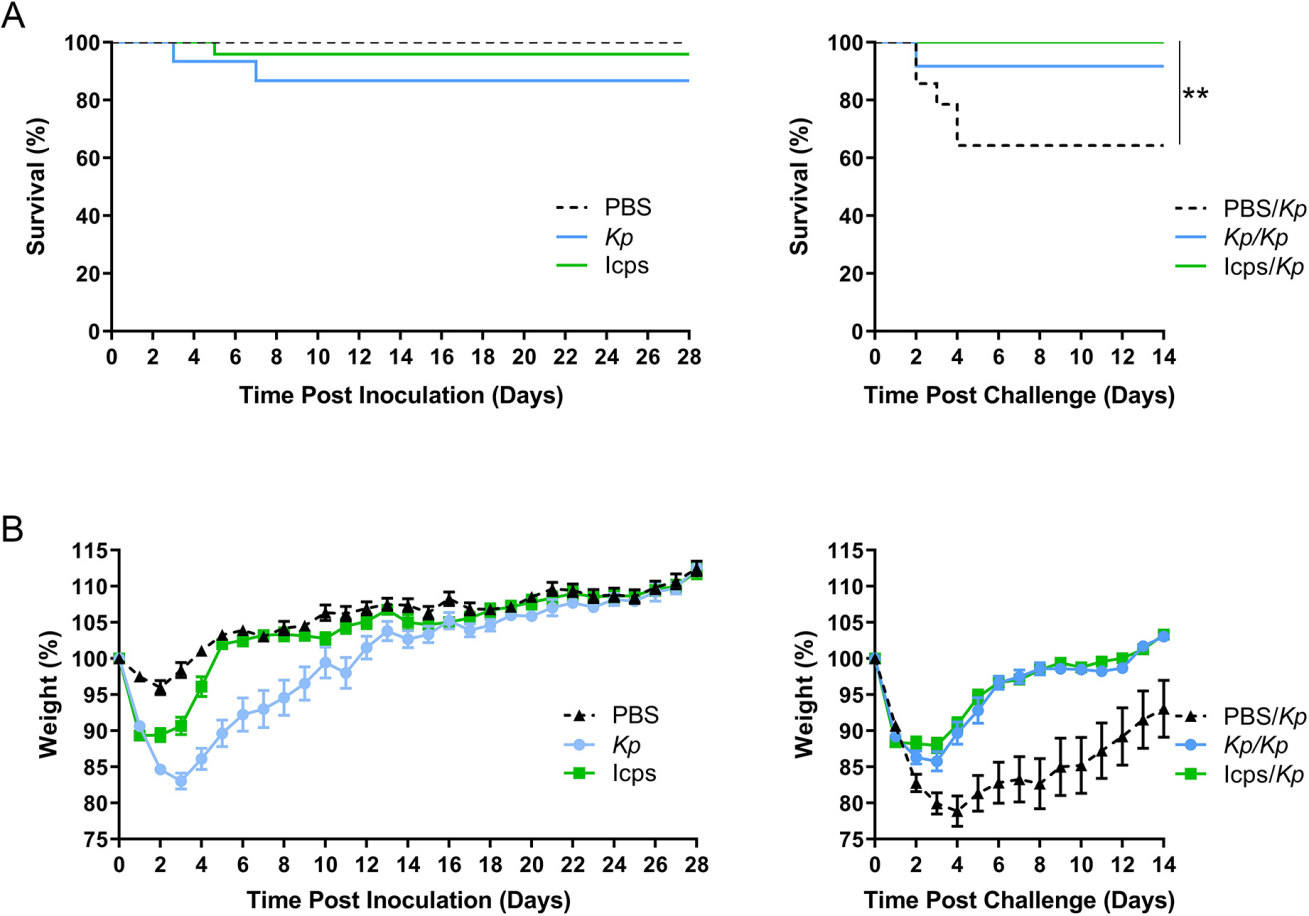
**Isolated capsule provides protection upon subsequent *Kp* challenge.** (A) Survival of mice inoculated with *Kp*, PBS or isolated capsule (Icps) over 28 days (left; *Kp n*=15, PBS *n*=15, Icps *n*=24) and over 14 days after challenge with *Kp* (right; *Kp/Kp n*=12, PBS*/Kp n*=14, Icps/*Kp n*=21). (B) Daily weights of mice inoculated with *Kp*, PBS or Icps over 28 days (left) and over 14 days after challenge with *Kp* (right). Data are shown as mean±s.e.m. and are combined from at least three independent experiments. **P<0.01; Mantel–Cox log-rank test.

Capsule has been the best-studied and most important antigen in protection from *K. pneumoniae* for decades (Bachman et al., 2015; Cryz et al., 1985; Lawlor et al., 2005, 2006; Schembri et al., 2005; Wu et al., 2009). To determine if *K. pneumoniae* capsule plays a critical role in adaptive immunity in our model, we isolated capsule from *K. pneumoniae* TOP52. Mice initially inoculated intratracheally with this isolated capsule (Icps), live *K. pneumoniae* or PBS were subsequently challenged with live *K. pneumoniae*. Most mice initially inoculated with PBS or Icps survived, and mortality in *K. pneumoniae*-inoculated mice (Fig. 5A) was consistent with previous experiments. Mice inoculated with Icps had significantly lower weights than PBS control mice (on days 1-4 post-inoculation) and significantly higher weights than mice inoculated with live *K. pneumoniae* (on days 2-9 post-inoculation) (Fig. 5B).

After challenge with *K. pneumoniae*, mice initially inoculated with Icps were protected from mortality, compared to mice initially inoculated with PBS (*P*=0.0031; Fig. 5A). Mice originally exposed to Icps also displayed significantly higher weights (on days 2-14 post-challenge), similar to those observed in *K. pneumoniae*-inoculated mice (Fig. 5B). These data demonstrate that a preparation of isolated capsule is sufficient to protect mice from subsequent *K. pneumoniae* challenge.

### Inoculation of mice with *K. pneumoniae* deficient in capsule, O-antigen or both confers protection from subsequent wild-type *K. pneumoniae* challenge

**Fig. 6.**
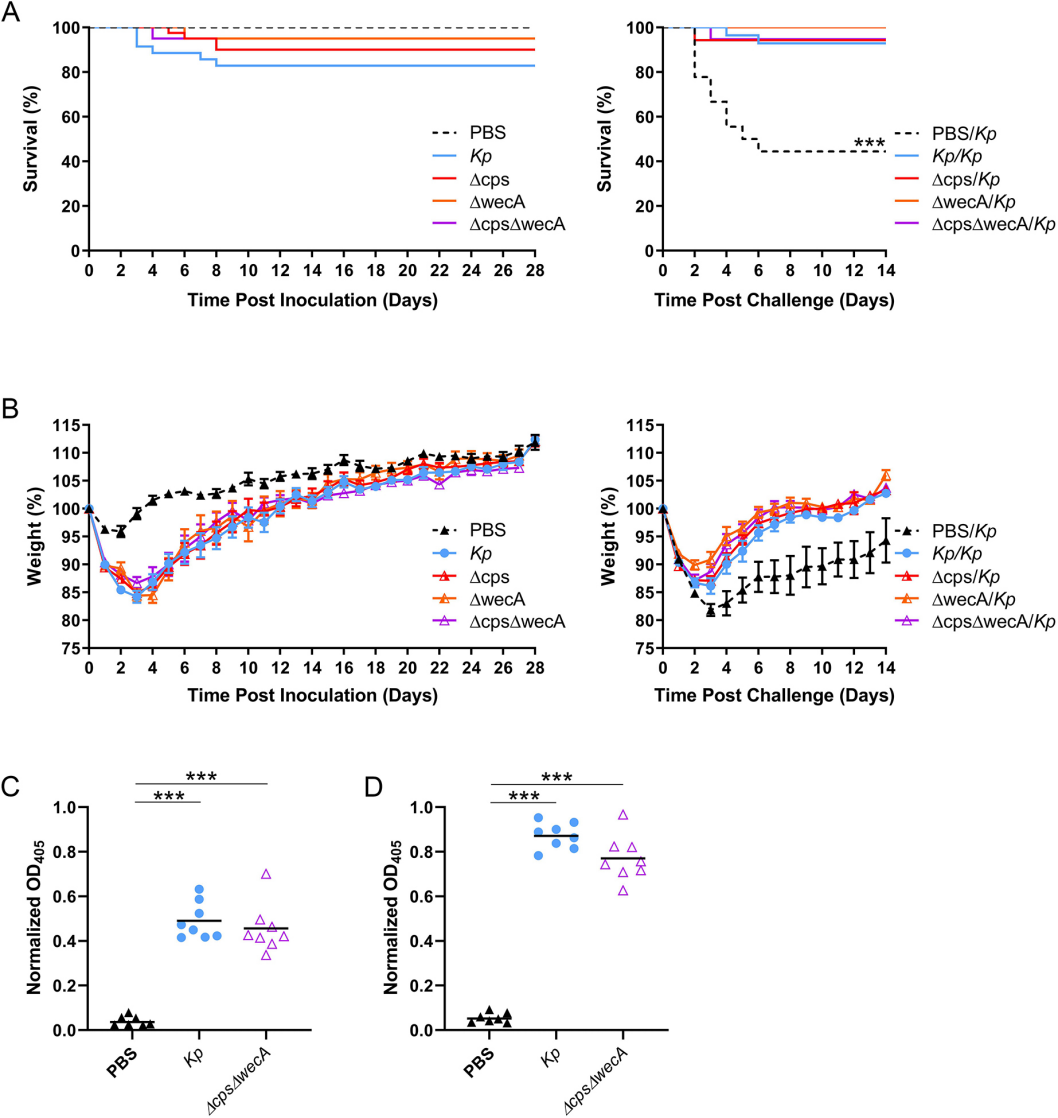
**Inoculation of mice with *Kp* deficient in capsule, O-antigen or both provides protection upon subsequent *Kp* challenge.** (A) Survival of mice inoculated with *Kp*, PBS, *Kp* lacking capsule (Δ*cps*), O-antigen (Δ*wecA*) or both (Δ*cps*Δ*wecA*) over 28 days (left; *Kp n*=35, PBS *n*=24, Δ*cps n*=40, Δ*wecA n*=20, Δ*cps*Δ*wecA n*=20) and over 14 days after challenge with *Kp* (right; *Kp/Kp n*=28, PBS*/Kp n*=18, Δ*cps/Kp n*=36, Δ*wecA/Kp n*=19, Δ*cps*Δ*wecA/Kp n*=19). (B) Daily weights of mice inoculated with *Kp*, PBS, Δ*cps*, Δ*wecA* or Δ*cps*Δ*wecA* over 28 days (left) and over 14 days after challenge with *Kp* (right). Weight data are shown as mean±s.e.m. and are combined from at least three independent experiments. (C) IgG ELISAs against whole *Kp* TOP52 using 1:50 diluted sera at 28 days post-inoculation. (D) IgG ELISAs against whole Δ*cps*Δ*wecA*. For ELISAs, each point represents the average of a single mouse serum run in triplicate. ***P<0.001 (for survival comparing PBS/*Kp* to all other groups, Mantel–Cox log-rank test; for ELISAs, Mann–Whitney *U*-test).

To determine if capsule was required for the protective phenotype, we constructed a capsular mutant in which a 16-kb region of the capsule synthesis operon from *wzi* onward was deleted in *K. pneumoniae* TOP52 (termed Δ*cps*). Additionally, O-antigen may play an important role in immunity to *K. pneumoniae* (Trautmann et al., 2004; Hsieh et al., 2014; Pennini et al., 2017; Rollenske et al., 2018). Therefore, we also constructed a mutant deficient in the *wecA* gene required for O-antigen production (Δ*wecA*), as well as a strain deficient in both capsule and O-antigen (Δ*cps*Δ*wecA*). Upon initial inoculation of mice with 10^7^ CFU of Δ*cps*, Δ*wecA* or Δ*cps*Δ*wecA*, we observed survival rates of 90-95% (Fig. 6A). After challenge of all survivors with wild-type *K. pneumoniae*, we observed survival rates of 94-100% among mice initially inoculated with these mutants (Fig. 6A). This is similar to survival of mice initially inoculated with wild-type *K. pneumoniae* and significantly higher than survival of PBS control mice upon *K. pneumoniae* challenge (*P*<0.0001 for each of the three mutant comparisons to wild type).

Mice initially inoculated with Δ*cps*, Δ*wecA* or Δ*cps*Δ*wecA* had weights similar to those of mice inoculated with wild-type *K. pneumoniae* and lower than those of PBS control mice (Fig. 6B). After challenge with wild-type *K. pneumoniae*, mice initially exposed to Δ*cps*, Δ*wecA* or Δ*cps*Δ*wecA* were protected from the weight loss observed in PBS control mice (on days 3-14 post-challenge), exhibiting weights similar to those in mice initially inoculated with wild-type *K. pneumoniae* (Fig. 6B).

Additionally, we analyzed sera of mice 28 days after infection with *K. pneumoniae* TOP52, Δ*cps*Δ*wecA* or PBS (mock infection). Enzyme-linked immunosorbent assays (ELISAs), performed using plates coated with whole-cell *K. pneumoniae* TOP52, demonstrated high levels of reactive IgG from mice infected with either TOP52 or Δ*cps*Δ*wecA* compared to mock-infected mice (*P*=0.0003 for both comparisons; Fig. 6C). Moreover, we tested sera in plates coated with whole-cell Δ*cps*Δ*wecA* and similarly found high levels of reactive IgG from mice infected with either TOP52 or Δ*cps*Δ*wecA*, compared to mock-infected mice (*P*=0.0003 for both comparisons; Fig. 6D). Together, these data suggest that antigens beyond capsular polysaccharide and O-antigen can contribute to protective immunity against *K. pneumoniae*.

## DISCUSSION

The emergence of hypervirulent *K. pneumoniae* strains and the development of extensive antimicrobial resistance make *K. pneumoniae* infections a worrisome and imminent threat to human health. Given this urgency, the development of vaccines or other immunotherapies to protect against *K. pneumoniae* infection is paramount, but is challenged by our incomplete understanding of adaptive immune responses to this pathogen. Here, we present a murine model of survivable pneumonia with live *K. pneumoniae* that enables exploration of natural adaptive immune response to *K. pneumonia*e infection of the respiratory tract. Mice that survive *K. pneumoniae* lung infection in our model were protected from morbidity and mortality upon subsequent challenge. While several fundamental observations regarding the nature of host response to *K. pneumoniae* have been made in models using heat-killed organisms (Chen et al., 2011; Amezcua Vesely et al., 2019), the present model permits the study of adaptive responses stemming from persistent bacterial-host interactions during live infection. Additionally, while most work in the field has relied on 24 h titer data as primary endpoints, this model also allows the use of morbidity and mortality to meaningfully evaluate the efficacy of potential therapies.

This murine model of immunity to *K. pneumoniae* can now be employed to further elucidate protective correlates of immunity. Recent work has demonstrated the contributions of T-helper 17 and tissue-resident memory T cells in the clearance of *K. pneumoniae* from the lung (Chen et al., 2011; Amezcua Vesely et al., 2019). Further experiments are underway in our model to identify specific host cell subsets required for protection. On the pathogen side, our most critical finding is that non-capsular antigens not only can participate in eliciting an adaptive response, but can also confer complete protection in the absence of capsule exposure. We have demonstrated serologic IgG responses to *K. pneumoniae* lacking both capsule and O-antigen, and although reactive IgGs have not been definitively correlated to protective immunity, they could conceivably be used to identify additional *K. pneumoniae* antigens. If appropriate non-capsular antigens can be identified, their inclusion in a potential vaccine might help to circumvent the limitation of serotype specificity that would accompany a capsule-based *K. pneumoniae* vaccine. Thus, we are currently examining the *K. pneumoniae* proteome in a reverse-vaccinology approach to identify bacterial antigens that could prove useful for incorporation into a multivalent vaccine to provide protection across multiple capsular serotypes.

Interestingly, we consistently observed an intermediate level of protection following inoculation of heat-killed organisms. This is important to note, given that others have studied adaptive responses using heat-killed inocula or extracellular vesicles in lieu of lethal live infections (Chen et al., 2011; Lee et al., 2015; Amezcua Vesely et al., 2019). We speculate that the nature and/or amplitude of immune responses to *K. pneumoniae* differ in hosts interacting with live versus heat-killed organisms or bacterial components, as observed in other infection systems (Bahjat et al., 2009; Moretti et al., 2017). It should be noted that we discerned full protection from isolated capsule inoculation alone compared to partial protection with heat-killed organisms, further suggesting a dependence of immune responses on the entire milieu of exposure at the host-pathogen or host-antigen interface. These observations are not trivial, as antigens found to be important for protection when delivered in isolation may not ultimately prove to be the most suitable vaccine candidates in the setting of dynamic host-pathogen interactions. Furthermore, the local environment of antigen delivery may play a large role in the nature of the subsequent immune response. While human vaccine delivery by intramuscular or subcutaneous injection offers some practical advantages (Miquel-Clopes et al., 2019; Neutra and Kozlowski, 2006), mucosal vaccines are already available for selected pathogens (e.g. influenza and polio) and are being studied in many infectious diseases. Using this murine model, additional work can compare and contrast the protection acquired via *K. pneumoniae* antigen exposure at the respiratory mucosa versus delivery of vaccine antigens at a distant site.

An intriguing finding of this work is the observation of perivascular and peribronchial lymphoid aggregates within the lungs of mice that survived *K. pneumoniae* infection. These lymphoid aggregates are morphologically consistent with previously described bronchus-associated lymphoid tissue (BALT) observed in murine lungs following several pulmonary infections (Chiavolini et al., 2010; Slight et al., 2013; Tan et al., 2019). In some models, the BALT functions as a set of tertiary lymphoid structures, enabling local T-cell priming and B-cell maturation. In *K. pneumoniae*-inoculated mice, the observed lymphoid aggregates could represent tertiary lymphoid structures required for the development of the robust pathogen-specific protection observed following challenge, or reflect less specific priming and activation (Halle et al., 2009). Future studies will interrogate the cellular composition and organization of these aggregates, as well as the relationship between proper formation of these structures and protection from re-infection.

This work introduces a system for studying adaptive immune responses to *K. pneumoniae* with the goal of informing future vaccine design. Correlates of protective immunity discovered and validated in this model system will ultimately require corroboration with human samples. While some promising *K. pneumoniae* vaccine candidates are already being studied (Hegerle et al., 2018; Feldman et al., 2019), these are unlikely to provide coverage across all strains capable of causing disease. By leveraging the present model, more conserved non-capsular (e.g. proteinaceous) antigens may be identified that could be combined with specific polysaccharide components to broaden *K. pneumoniae* vaccine coverage.

## MATERIALS AND METHODS

### Bacterial strains, mutant construction and culture conditions

*K. pneumoniae* strain TOP52 and mutants derived from this parent wild-type isolate were used for all experiments. Previous capsule K-typing by the Statens Serum Institut, using historical sera, identified this strain as capsular type K6. However, using the Institut Pasteur *Klebsiella* Sequence Typing Database and sequencing data, this isolate was found to be sequence type 152 and carry *wzi* allele 150, which corresponds to associated KL types of KL163, KL27 and KL46 (Johnson et al., 2014; Rosen et al., 2008; Wylie et al., 2019). A modified lambda Red recombinase protocol was utilized to construct Δ*cps* (lacking a 16-kb region of *cps* operon starting from *wzi*) and Δ*wecA* using pKD46s (Bachman et al., 2015). Linear DNA required for recombination events was amplified from pKD4 using the following primers: cpsF, 5′-ATGATAAAAATTGCGCGCATTGCCGTGACGTTGGGTTTGCTTTCCTCACTGGGAGCCCAGGTGTAGGCTGGAGCTGCTTC-3′; cpsR, 5′-CTCTGCCAATCCTGTACTGACCTATAATGCCTAACAGGAATTATAAAATTAATTGCAAAGCATATGAATATCCTCCTTAG-3′; wecAF, 5′-GCTTGTGCTCCCGGTAATGGTTGAGTCATCACATCCCGTGTAGGCTGGAGCTGCTTC-3′; and wecAR, 5′-CGCTATACTTCCCGGATTAACTATGCTGAGAGCACATGCGCATATGAATATCCTCCTTAG-3′. Kanamycin cassettes were subsequently removed using the helper plasmid pCP20 encoding FLP recombinase (Datsenko and Wanner, 2000). To make Δ*cps*Δ*wecA*, the protocol for constructing the *wecA* mutant was applied to Δ*cps.* All mutants were confirmed by sequencing of amplicons generated by PCR using the following primers: cps checkF, 5′-GGGTAAATGTACTTGCCTCGCCG-3′; cps checkR, 5′-AACACTCTGCCAATCCTGTACTGACC-3′; wecA checkF, 5′-GGTGTACACCAGCACGATGGC-3′ and wecA checkR, 5′-CCAGAGACAGAGAAAGCG-3′.

For preparation of murine inocula, bacteria were grown statically in 20-ml cultures at 37°C for 16 h in Luria-Bertani (LB) broth. Cultures were centrifuged at 8000 ***g*** for 10 min, and bacteria were subsequently resuspended in sterile PBS and diluted to the desired inoculum concentration by measuring optical density at 600 nm (OD_600_). Inocula were verified by serial dilution and plating. For heat-killed TOP52 experiments, inocula were incubated at 60°C for 30 min; plating of these aliquots confirmed the lack of live bacteria.

### Mouse infections

All animal procedures complied with ethical regulations for animal testing and research and were approved by the Institutional Animal Care and Use Committee at Washington University School of Medicine. Female C57BL/6J mice (Jackson Laboratories, Bar Harbor, ME, USA) or RAG1^−/−^ mice (B6.129S7-*Rag1^tm1Mom^*/J) were 7-8 weeks old at the onset of all experiments. For initial inoculations, an intratracheal administration procedure was adapted from those previously described (Deng et al., 2004). Briefly, each mouse was anesthetized with inhaled isoflurane, and the trachea exposed through surgical dissection. Inoculum (20 μl containing either 1-2×10^7^ CFU, sterile PBS or isolated capsule) was injected intratracheally using a 30-gauge, caudally directed needle. Overlying tissues were replaced and skin was closed using Vetbond (3 M Animal Care Products, St Paul, MN, USA). Mice received 1 mg/kg of buprenorphine SR subcutaneously for pain control. Mice were assessed for mortality and weighed daily. After 28 days, the majority of surviving mice (now aged 11-12 weeks) were challenged with 1-2×10^7^ CFU TOP52 using the same method as above. Mortality and weight changes were assessed for an additional 14 days prior to sacrifice. In some instances, mice were sacrificed at 28 days post-inoculation or at other noted time points to perform histopathologic analyses or measure bacterial titers, as described below. For ELISAs, mice were inoculated via oropharyngeal aspiration as described below.

### Murine organ titers and histology

Murine organs (lungs, spleens) were harvested from surviving mice at the conclusion of each experiment, or from mice at predetermined time points throughout the experiments. The right lung was prepared for bacterial titer; the left lung was processed for histology. Organs for titer were homogenized in sterile PBS via a Bullet Blender (Next Advance, Averill Park, NY, USA) for 5 min. A 200-μl aliquot was removed from the 1-ml homogenate, serially diluted and plated on LB agar. Organs for histology were washed in PBS, fixed in 10% neutral buffered formalin, dehydrated in ethanol and embedded in paraffin; 5-μm sections were stained with Hematoxylin and Eosin. Images were obtained using an Olympus DP25 camera and BX40 light microscope.

### Capsule isolation

Capsular material was isolated from *K. pneumoniae* TOP52 as previously described (Zamze et al., 2002), with some modifications. Briefly, bacteria were cultured overnight at 37°C, shaking at 90 rpm in 3 l LB broth. The bacteria were centrifuged at 8000 ***g*** for 20 min and decanted, and the pellet was resuspended in 120 ml deionized water. The resuspended pellet was heated in a 100°C water bath for 15 min and then cooled to room temperature (RT). Then, 480 ml acetone was added to a final concentration of 80% (v/v) and stirred gently at 4°C overnight to precipitate capsular material. The precipitate was decanted, air dried for 48 h and lyophilized. Yield of isolated capsular product was quantified by uronic acid assay as previously described (Rosen et al., 2015). Mice were inoculated with 25-50 μg lyophilized capsular product resuspended in 20 μl PBS.

### ELISAs

Mouse sera used for ELISAs were obtained from mice at 28 days post-oropharyngeal aspiration (Kudva et al., 2011) with 50 μl of 1-2×10^8^ CFU of *K. pneumoniae* TOP52, Δ*cps*Δ*wecA* or sterile PBS. Relative IgG levels were determined for individual mice in triplicate by a method similar to one previously described (Cox et al., 2015). Briefly, 96-well flat-bottom plates were coated with ∼5×10^6^ CFU in 100 μl per well of live *K. pneumoniae* TOP52 or Δ*cps*Δ*wecA*. These bacterial strains were grown statically overnight in LB broth at 37°C, centrifuged at 8000 ***g*** for 10 min, resuspended in sterile PBS (OD_600_=∼0.85) and diluted 1:10 in carbonate coating buffer prior to coating. Inocula were verified by serial dilution and plating. After incubating overnight at 4°C, plates were washed 3× with PBS with 0.05% Tween-20 (0.05% PBST), blocked with 5% bovine serum albumin for 1 h and washed 3× with 0.05% PBST at RT. Mouse sera were diluted 1:50 in 0.05% PBST, and 50 μl was applied per well and incubated overnight at 4°C. Plates were then washed 5× with 0.05% PBST before 100 μl of 1:5000 horseradish peroxidase-conjugated anti-mouse IgG antibodies (GE Healthcare #NA931) in 0.05% PBST were added to each well for 1 h at RT. Plates were subsequently washed 5× with 0.05% PBST, developed using ABTS peroxidase substrate (Seracare #5120-0032) for 30 min at RT and stopped with 1% sodium dodecyl sulfate. The optical density of the reaction was recorded at 405 nm using a Synergy 2 Multi-Mode Microplate Reader (BioTek, Winooski, VT, USA) and corrected by subtracting the negative control well values prior to analysis.

### Statistical analysis

For Kaplan–Meier survival analyses, the Mantel–Cox log-rank test was used to determine differences in survival between two groups. Comparisons between two groups of normally distributed continuous variables (mouse weights) were analyzed using Student's *t*-tests with Holm–Sidak correction for multiple comparisons. For values not definitively normally distributed (organ titers, ELISA values), the Mann–Whitney *U*-test was used. All tests were two-tailed, and *P*-values <0.05 were considered significant. Analyses were performed using GraphPad Prism 8.02.

## Supplementary Material

Supplementary information
