# Supplementary material for: A murine model demonstrates capsule-independent adaptive immune protection in survivors of Klebsiella pneumoniae respiratory tract infection
Source: Dis Model Mech. 2020 Mar 26;13(3):dmm043240. doi: 10.1242/dmm.043240 (PMC7104859; doi:10.1242/dmm.043240)
Supplement: Supplementary information [file dmm-13-043240-s1.pdf]

SUPPLEMENTAL FIGURES

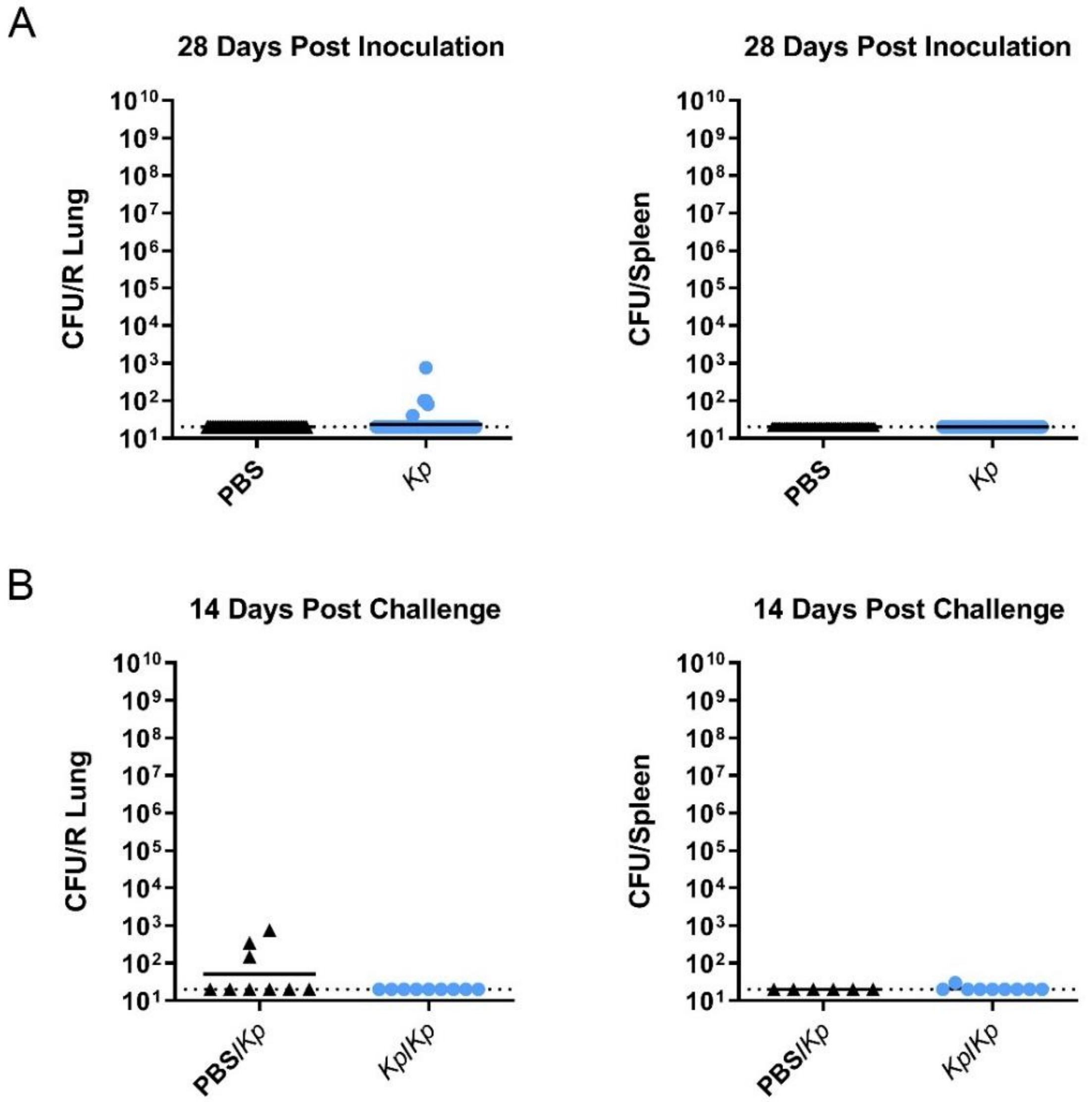

Figure S1. Survivors of *K. pneumoniae* TOP52 inoculation and challenge demonstrate low or absent lung and spleen bacterial burden. A. The majority of mice have undetectable or low bacterial titers in their right lung (left panel) or spleen (right panel) 28 days post inoculation with PBS (black) or *Kp* (blue). B. Similarly, the majority of mice have undetectable or low bacterial titers in their right lung (left panel) or spleen (right panel) 14 days post challenge with *Kp*. Data are combined from at least 3 independent experiments. Each symbol represents one animal, short bars represent geometric means of each group, and full dotted horizontal lines represent limits of detection.

Disease Models & Mechanisms • Supplementary information

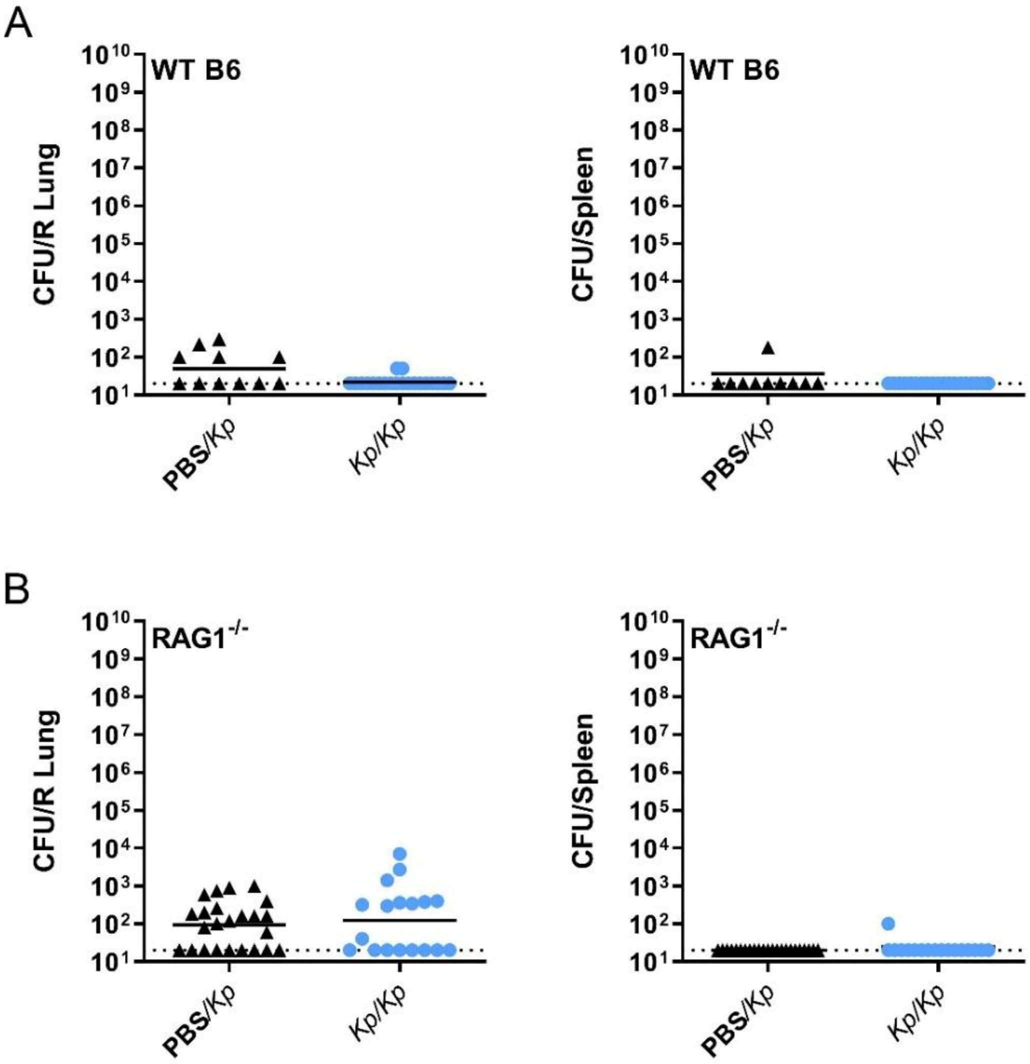

Figure S2. Both wild-type and RAG1$^{-/-}$ survivors of *K. pneumoniae* TOP52 challenge demonstrate low or absent lung and spleen bacterial burden. A. The majority of wild-type C57BL/6 mice have undetectable or low bacterial titers in their right lung (left panel) and spleen (right panel) 14 days post challenge with *Kp.* B. RAG1$^{-/-}$ survivors also have low bacterial titers 14 days post challenge with *Kp* in their right lung and spleen (albeit a higher proportion of mice had detectable lung colonization compared to wild-type C57BL/6 mice at this timepoint). Data are combined from at least 3 independent experiments. Each symbol represents one animal, short bars represent geometric means of each group, and full dotted horizontal lines represent limits of detection.

Disease Models & Mechanisms • Supplementary information
